# The Frequency of Hepatitis C and its Risk Factors Among Health Care Providers at Tehsil Headquarter Hospital, Hasilpur, Pakistan

**DOI:** 10.7759/cureus.3176

**Published:** 2018-08-21

**Authors:** Uffan Zafar, Ammar Hasan, Bilal Aslam, Zubair Khalid, Muhammad Usman Baig, Saba Akram

**Affiliations:** 1 Radiology Department, Bahawal Victoria Hospital, Quaid-E-Azam Medical College, Bahawalpur, PAK; 2 Lahore Medical and Dental College, Lahore, PAK; 3 Bahawal Victoria Hospital, Quaid-E-Azam Medical College, Bahawalpur, PAK; 4 Medical Ward, Bahawal Victoria Hospital, Quaid-E-Azam Medical College, Bahawalpur, PAK; 5 Medicine, Bahawal Victoria Hospital, Bahawalpur, PAK

**Keywords:** hepatitis c, healthcare workers, risk factors, frequency of hepatitis c

## Abstract

Introduction

Hepatitis C has emerged as a big challenge for Pakistan. Health care providers are at increased risk of being infected due to tremendous exposure.

Aim

The purpose of this study was to find the prevalence of hepatitis C, its risk factors especially its occupational risk factors and preventive measures practiced by health care providers of Tehsil Head Quarter Hospital, Hasilpur, Pakistan.

Materials and methods

Blood samples were collected, and rapid chromatography strips were used for diagnostic purpose. A questionnaire was used for data collection. After analyzing the data, results were summarized.

Results

The prevalence of Hepatitis was 5.17%. Those who used gloves were 67.24%. A history of needle stick injury was present in 47.41%, and 49.13% knew about the spread of Hepatitis C Virus (HCV). Just 18.96% knew about the treatment plan, and 19.83% had attended a workshop on preventive measures of infectious diseases in healthcare settings.

Conclusion

The frequency of HCV among health care providers is high. Awareness programs and training workshops should be mandatory to increase knowledge about hepatitis C prevention. It will decrease the incidence of hepatitis C infection among health care workers.

## Introduction

Hepatitis C Virus (HCV) infection is one of the leading causes of morbidity and mortality in under-developed countries like Pakistan. According to an estimate, 6% of Pakistan’s total population suffers from HCV infection which makes a total of 10 million people [1]. There are 240,000 newly diagnosed cases of Hepatitis C every year in Pakistan [2]. HCV infection can cause acute hepatitis, chronic liver disease, and hepatocellular carcinoma. Hepatitis kills 1.45 million people every year in the world [3]. The most important risk factors for Hepatitis C transmission are unscreened blood transfusions and unsafe drug injections [4]. Other risk factors are polygamous sexual relationships, razor sharing, tattooing and piercing, needle-stick injuries, contaminated medical equipment, dental procedures by quacks and blood spillage in health care settings [5, 6]. Sharing of personal items like toothbrushes is also a possible risk factor for HCV transmission, but further researches are needed to prove it [7].

Health care providers (HCPs) are considered to be the high-risk individuals because they are all the times exposed to contaminated sharp devices and get infected by multiple infectious diseases including hepatitis C. The risk factor for HCV transmission among healthcare workers at their workplace is a percutaneous injury like a needle stick injury [8]. The rate of acquiring infection is much higher among HCPs of primary and secondary health care centers. The overuse of injection practices, the lack of training workshops, the lack of knowledge about prevention and the lesser number of safety equipment make them more vulnerable. Not many studies are available on the frequency as well as determinants of Hepatitis C among HCPs working in primary and secondary health care centers in Pakistan. This data will help in creating awareness among HCPs about Hepatitis C, and it will help them to practice preventive measures. This study will also urge higher authorities to provide safety equipment, to do repeated screening and to arrange training workshops for HCPs. This study is designed to collect information regarding frequency and determinants of HCV in health care personnel and their knowledge about treatment and prevention at Tehsil Head Quarter (THQ) hospital, Hasilpur.

## Materials and methods

It was a cross-sectional study that was conducted among health personnel of THQ Hospital, Hasilpur for the period of four months, from September 2017 to December 2017. The sample size was 116, and the universal sampling technique was used. All the willing health care workers of THQ Hospital, Hasilpur were included. The non-willing health care workers and persons other than health care workers were excluded. Informed verbal and written consent was taken before the interview. The respondents were informed about the purpose of the study. The confidentiality of all the information was ensured and maintained. The data collection tool was a semi-structured interview questionnaire containing both open-ended and close-ended questions. These questions assessed their knowledge about HCV transmission, the standard precautionary measures recommended by the WHO, and the treatment plan. Details about personal medical and surgical history were obtained. We also took the family history that mainly included if the spouse or any other family member were suffering from Hepatitis C. We inquired if there were any workshops for educating and training health care workers against their occupational exposure to the bloodborne pathogens. They were asked if there was any accidental needle prick or exposure to the other risk factors like blood transfusions and dental work done by quacks. We took information about the provision of needle cutters. The researchers visited THQ Hospital, Hasilpur. They collected blood samples and tested them for Hepatitis C via rapid chromatography strip technology. The questionnaires were filled. Prior permission for collection of the sample was taken from the Medical Superintendent of the hospital. For data entry and analysis we used SPSS 17.0 (SPSS Inc., Chicago, IL) and Microsoft Office Word.

## Results

**Figure 1:**
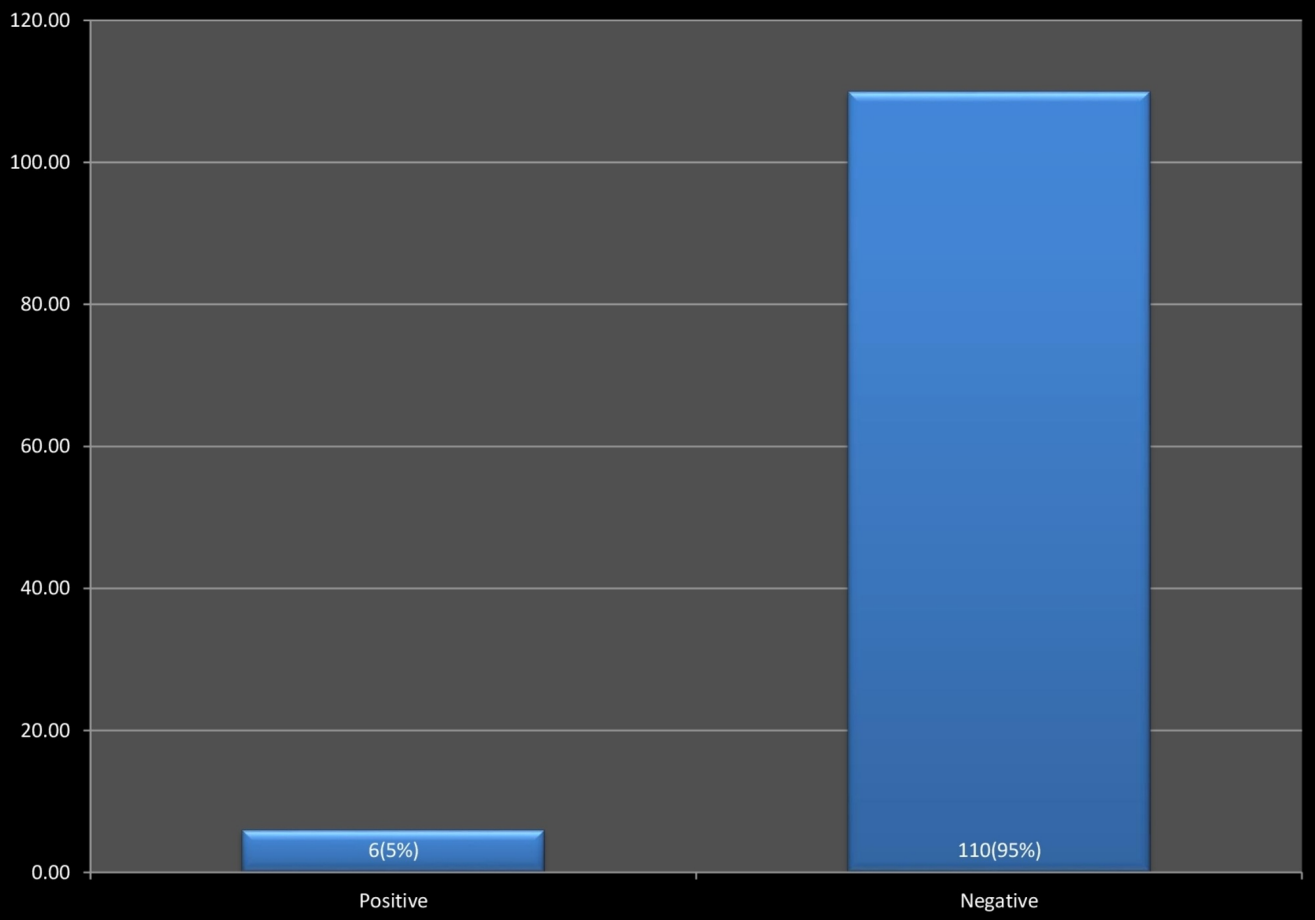
Frequency of hepatitis C in health care providers.

The average age of HCPs was 39 years. Seventy percent of the HCPs were male, and 30% were females. Doctors were 31.5%, and the paramedical staff was 68.5%. Singles were 6.9%, and 93.1% were married. Figure 1 describes the prevalence of hepatitis C infection among health care providers.

**Figure 2:**
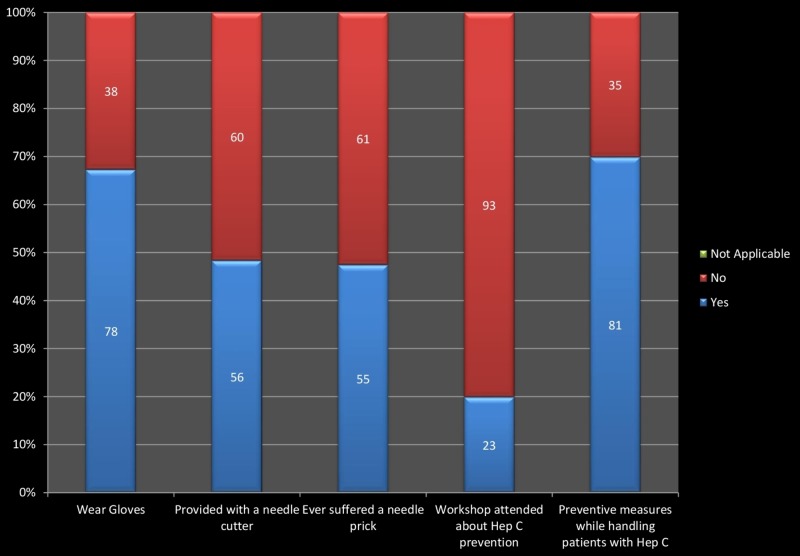
Preventive measures of hepatitis C practiced by health care providers and its risk factors at workplace.

The knowledge about the spread of HCV was 49.13% among HCPs. The knowledge about the existence of an effective treatment plan for HCV is poor (18.96%) among the health care workers. The majority wears gloves, but they do not use a needle-cutter. The majority has not attended any workshop on hepatitis prevention, and they also do not observe preventive measures while handling patients with Hepatitis C. More than 45% of them suffered a needle prick at least once. Figure 2 describes the frequency of health care workers practicing the different preventive measures and their frequency of having different risk factors for Hepatitis C at the workplace.

**Figure 3:**
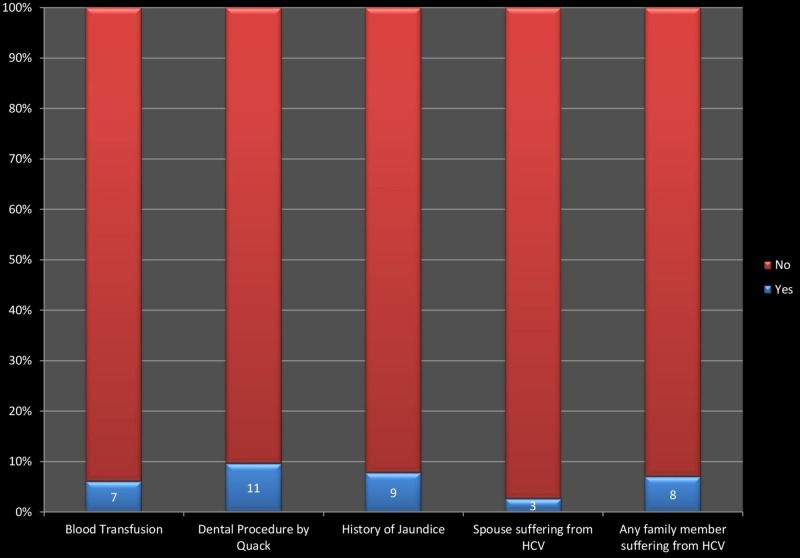
Risk factors associated with medical, surgical and family history of health care providers.

There is a mandatory screening of all the HCPs for HCV before the start of their job. There is no schedule for regular screenings. Just a single HCP had a tattoo, and a single HCP had multiple sexual partners. Medical and surgical history of health care providers is a risk factor for Hepatitis C transmission. Nose or ear piercing is the most common risk factor found in all females. After this, the dental procedure by a quack is the most common risk factor followed by a family member suffering from HCV, blood transfusion history and spouse suffering from HCV. Figure 3 provides details of these risk factors among healthcare providers.

## Discussion

We found that the prevalence of Hepatitis C among HCPs was 5.17%. It was more than that of another study conducted in Pakistan having a prevalence of 4.13% among HCPs [8]. It was even more than the prevalence of HCV among HCPs as shown by a study conducted in India [9]. This difference is because the HCPs adopt better preventive measures in tertiary health-care centers. In contrary to that, we performed our study in the secondary health-care center having lesser trained HCPs. According to a study, the HCV prevalence in healthy adults in the general population of Pakistan was 3.0% [10].

We found that nearly half of the HCPs were aware of the spread of HCV but only 18.96% had the knowledge about an effective treatment plan of Hepatitis C. The findings of other studies had a better scenario where 42.5% and 31.4% of HCPs knew about effective treatment plan of HCV [11, 12]. Safety measures adopted by HCPs have a significant role in preventing infections. More than half (67.24%) of HCPs used gloves while handling patients. Another study shows 62.7% of the HCPs knew the importance of using gloves for the prevention of HCV infection [13]. The result of our study is even better than a study conducted in Pakistan where 46.7% of HCPs used gloves [12].

In our study, a needle cutter was available to 48.28% of the HCPs and about half of the HCPs (47.41%) had a history of needle stick injury (NSI). In another study, a needle cutter was available to 93.5% of the HCPs and a history of NSI was present in 31% of the HCPs. That study shows that we can prevent more than 80% of the needle stick injuries with the use of safe needle devices and the training of workers [14].

Moreover, the non-occupational risk factors for HCV are also common among HCPs. In our study, 6.03% had a history of blood transfusion. A history of a dental procedure by a quack was present in 9.48%. Nose or ear piercing was the most common risk factor found in all females. Mostly quacks performed it without undertaking aseptic measures in non-professional settings. Just a single HCP had a tattoo. Some health care workers had a history of jaundice, razor sharing, and spouse or any other family member suffering from HCV. None of the HCPs had a history of intravenous (IV) drug abuse. A single HCP had multiple sexual partners. The higher prevalence of HCV among HCPs of our study may be attributed to these risk factors as well. Another study describes these non-occupational risk factors among HCPs. In that study, 4.8% had a history of blood transfusion and 8.2% had a tattoo. A history of dental procedure was present in 44.9%. No HCP was IV drug abuse, and none of them had multiple sexual partners.

Our study showed only 19.83% of the HCPs attended a training workshop on preventive measures of HCV. In a study at Nishtar Hospital (a tertiary care center in Pakistan), 25.7% of HCPs had attended a training workshop on HCV [12]. The health department should organize continuous training workshops for prevention of HCV in all the primary and secondary health care centers. In case of accidental exposure, an efficient system should be there for reporting. The affected health care workers should receive treatment. The use of preventive measures should be according to the standards laid down by the World Health Organisation (WHO). The higher authorities should ensure the provision and the use of safety equipment such as safe needle devices. The research had some limitations. In the study, there was no control arm representing the general population to compare its frequency of HCV with that of the health care providers. Moreover, our study was descriptive. The analytical studies determining the significance of each of the risk factors are required.

## Conclusions

The frequency of HCV among health care providers is high. The knowledge about the prevention and treatment is poor among them. The use of preventive measures is poor. Safety equipment such as safe needle devices should be available. There should be mandatory training workshops for prevention of HCV in every hospital. These workshops will help in decreasing the frequency of HCV and all other nosocomial infections.

## References

[1] Hussain S, Patrick NA, Shams R (2010). Hepatitis B and C prevalence and prevention awareness among health care workers in a tertiary care hospital. Int J Pathol.

[2] Alaei K, Sarwar M, Juan SC, Alaei A (2016). Healthcare and the preventable silent killer: the growing epidemic of hepatitis C in Pakistan. Hepat Mon.

[3] Zoulim F, Liang TJ, Gerbes AL (2015). Hepatitis C virus treatment in the real world: optimising treatment and access to therapies. Gut.

[4] Manns MP, Buti M, Gane E, Pawlotsky J-M, Razavi H, Terrault N, Younossi Z (2017). Hepatitis C virus infection. Nat Rev Dis Primers.

[5] Zia A, Ullah I, Ali S (2015). Prevalent risk factors of HCV transmission in health care workers (HCWS) in Pakistan. Int J Pharm Pharm Sci.

[6] He Y, Zhang J, Zhong L (2011). Prevalence of and risk factors for hepatitis C virus infection among blood donors in Chengdu, China. J Med Virol.

[7] Lock G, Dirscherl M, Obermeier F (2006). Hepatitis C - contamination of toothbrushes: myth or reality?. J Viral Hepat.

[8] Khan S, Attaullah S, Ayaz S (2011). Molecular epidemiology of HCV among health care workers of Khyber Pakhtunkhwa. Virol J.

[9] Shah DK, Jain SS, Khot AA, Gharat AR, Rajadhyaksha GC, Rathi PM (2017). Low prevalence of hepatitis B and C infections among the healthcare workers despite low vaccination coverage for hepatitis B in Mumbai. Indian J Med Sci.

[10] Ali SA, Donahue RM, Qureshi H, Vermund SH (2009). Hepatitis B and hepatitis C in Pakistan: prevalence and risk factors. Int J Infect Dis.

[11] Shindano TA, Bahizire E, Fiasse R, Horsmans Y (2017). Knowledge, attitudes, and practices of health-care workers about viral hepatitis B and C in South Kivu. Am J Trop Med Hyg.

[12] Ali MF, Ahmad A, Khan MM (2018). Frequency of hepatitis C and awareness of its health hazards among paramedical staff. Pak J Med Health Sci.

[13] Alnoumas SR, Enezi F, Isaeed M, Makboul G, El-Shazly MK (2012). Knowledge, attitude and behavior of primary health care workers regarding health care-associated infections in Kuwait. Greener J Med Sci.

[14] Akeem BO, Abimbola A, Idowu AC (2011). Needle stick injury pattern among health workers in primary health care facilities in Ilorin, Nigeria. Acad Res Int.

